# Mechanical Performance of Composite Laminates and Sandwich Panels Upcycled from Carbon and Glass Fiber Prepreg Scraps

**DOI:** 10.3390/ma19204359

**Published:** 2026-10-16

**Authors:** Jianqun Hao, Emma Declercq, Konstantinos Giannadakis, Danny Van Hemelrijck, Kalliopi-Artemi Kalteremidou

**Affiliations:** 1 Research Group Mechanics of Advanced Materials and Structures (M2S), Department of Mechanics of Materials and Constructions, Vrije Universiteit Brussel (VUB), 1050 Brussels, Belgium; 2 Orfit Industries, 2110 Wijnegem, Belgium

**Keywords:** uncured prepreg scrap, reuse, mechanical performance retention, failure mechanisms

## Abstract

Composite manufacturing generates large quantities of uncured prepreg scraps that retain continuous fibers and uncured resin, providing an opportunity for high-value reuse. Unlike previous studies that have largely focused on coupon-level samples with relatively simple scrap layouts, the present work comparatively evaluates three patch-assembly configurations in both glass and carbon fiber epoxy systems, extends the approach to foam-core sandwich structures, and assesses its structure-level feasibility through a head–neck board demonstrator. Tensile and flexural properties were evaluated against virgin reference laminates, followed by three-point bending tests on sandwich beams. Failure mechanisms were analyzed using stress-based calculations and optical microscopy. Results showed scrap assembly reduced tensile strength because of ply-joining interfaces, whereas flexural performance was less affected, particularly for rectangular patch assemblies. Among the investigated geometries, rectangular patches consistently achieved the highest mechanical performance retention for both fiber systems. In sandwich structures, glass fiber specimens failed predominantly by foam-core shear, while carbon fiber specimens exhibited local skin wrinkling caused by elastic instability. A head–neck board demonstrator manufactured from rectangular scrap-assembled carbon fiber sandwich panels retained 77% of the bending capacity of a commercial reference while exceeding the load-bearing requirement for clinical use. These results demonstrate that direct reuse of uncured prepreg scraps is a technically viable route for producing structural composite components and advancing circular composite manufacturing.

## 1. Introduction

The rapid growth in the use of composite materials has been accompanied by a significant increase in associated waste streams, which can be broadly classified into manufacturing-stage prepreg waste and end-of-life composite structures [1,2]. Prepreg waste arises predominantly from manufacturing operations, including cutting offcuts, end-of-roll remnants, and the disposal of out-of-specification or expired prepreg rolls [3]. From the perspective of matrix chemistry, prepreg systems can be broadly divided into thermoplastic and thermoset composites. Thermoplastic matrices can generally be reheated and reshaped, whereas thermoset matrices undergo irreversible crosslinking during curing and cannot be remelted or reformed after full cure. Before curing, however, thermoset prepregs remain processable and can be shaped, stacked, consolidated, and co-cured into laminates and structural components [4,5]. Consequently, uncured thermoset prepreg scraps represent a particularly valuable manufacturing waste stream because they retain both their continuous fiber architecture and uncured resin system, allowing direct reuse without first recovering the reinforcement from a fully cured composite [6]. This retained material quality makes direct prepreg reuse a promising high-value circular manufacturing strategy, while reducing the environmental impacts associated with conventional recycling processes [7,8].

Despite this clear potential, uncured thermoset prepregs are commonly discarded due to practical challenges such as handling complexity, reduced dimensions, and irregular geometries, leading to significant economic losses and environmental burdens [9]. As composite production continues to expand, the development of robust and scalable strategies for the reuse of uncured prepreg waste is therefore becoming increasingly essential.

Currently, most established work focuses on managing end-of-life composite waste, where the aim is to recover fibers from fully cured components through processes such as mechanical milling, pyrolysis, and solvolysis [10,11,12]. These methods have been extensively investigated because they address large volumes of composite waste generated after service life, particularly in the aerospace, automotive, and wind energy sectors. However, manufacturing-stage waste is also a critical concern, as uncured prepreg offcuts can account for up to 40% of the total prepreg used in primary structures [13]. Moreover, this waste is generated continuously during production, leading to substantial accumulation if not addressed. Its management is probably more immediate in practice than the recycling of end-of-life composite structures, which reach disposal only after long service lives [14].

Reuse of uncured prepreg scrap has been explored through several distinct processing routes, especially for carbon fiber thermoset polymer prepregs. One approach is chip-based processing, where prepreg scraps are chopped into small pieces and consolidated by compression molding; see typical works in [15,16,17,18,19]. This process produces discontinuous composites with quasi-isotropic properties, which is suitable for molding complex geometries and manufacturing secondary structures. A second major route is the ply-level assembly of regular scrap patches, which are stacked and cured to form new laminates. This approach has been further extended by hybridizing scrap-based plies with virgin plies, thereby broadening their applicability to structural and semi-structural components, as demonstrated in [20,21]. Compared with chip-based processing, ply-level assembly is advantageous for applications demanding higher mechanical performance, because the intact scrap pieces maintain fiber continuity and promote performance retention. Additionally, this route enables controlled fiber orientation and stacking, producing laminate-like behavior. However, the scrap-assembled laminates are sensitive to the geometry and arrangement of the scrap patches, as discontinuities at patch boundaries can lead to stress concentrations, weak resin-rich regions, or misalignment.

While several studies have evaluated the mechanical performance of scrap patch-assembled laminates, these investigations are largely restricted to simple strip- or square-shaped carbon fiber prepreg patches, as reported in [17,18,22,23]. The influence of different scrap geometries on the mechanical properties of different prepreg systems has not been systematically investigated. Furthermore, most work is confined to flat laminates, with very little data available for sandwich structures incorporating scrap-derived skins, and practical demonstrators based on such materials are rarely reported.

Previous studies on the direct reuse of uncured prepreg scrap have predominantly focused on carbon fiber systems, relatively simple strip- or square-based scrap layouts, and monolithic laminates. The present work extends this research by comparatively evaluating rectangular, diamond, and hexagonal patch-assembly configurations in both glass and carbon fiber epoxy systems, assessing their application as skins in foam-core sandwich structures, and demonstrating component-level feasibility using a head–neck immobilization board. In addition to mechanical performance retention, the study examines the associated laminate and sandwich failure behavior.

To investigate how scrap geometry influences structural performance, the scraps were cut into three representative geometries—rectangular, diamond, and hexagonal patches—and assembled at the ply level to form composite laminates and sandwich skins. The resulting materials were evaluated through tensile and flexural testing. Fracture morphology analysis was performed to examine the failure mechanisms associated with different scrap geometries. The practical applicability of the reuse approach was demonstrated through the fabrication of a head–neck immobilization board for radiotherapy treatment.

## 2. Materials and Methods

### 2.1. Assembling of Prepreg Scraps

The woven (2/2 twill) carbon and glass fiber epoxy prepregs were provided by Mitsubishi Chemical Group (Heinsberg, Germany). The material properties of the used prepreg tapes are shown in Table 1.

**Figure 1:**
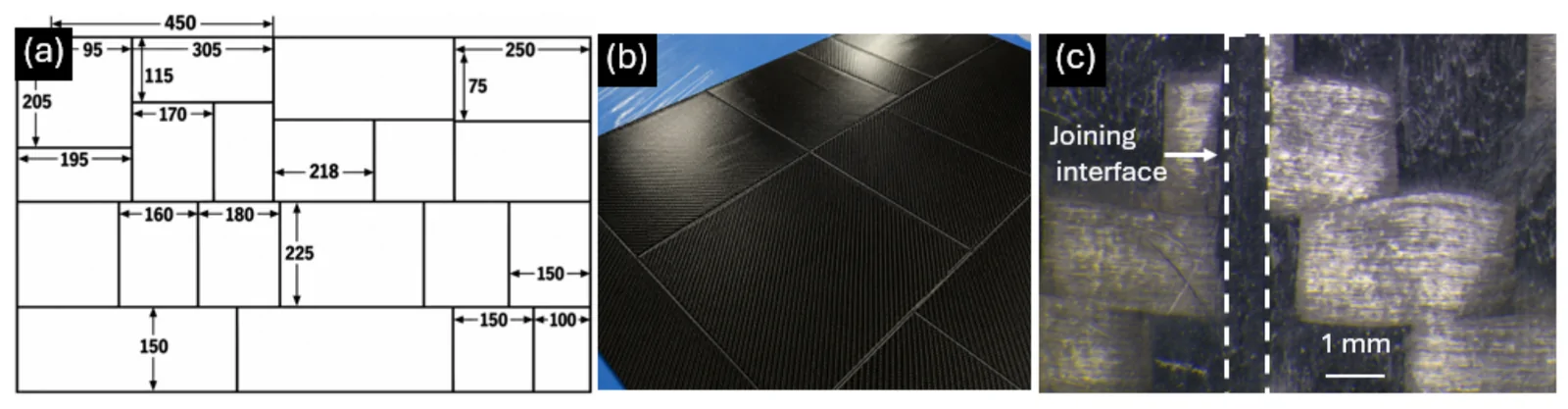

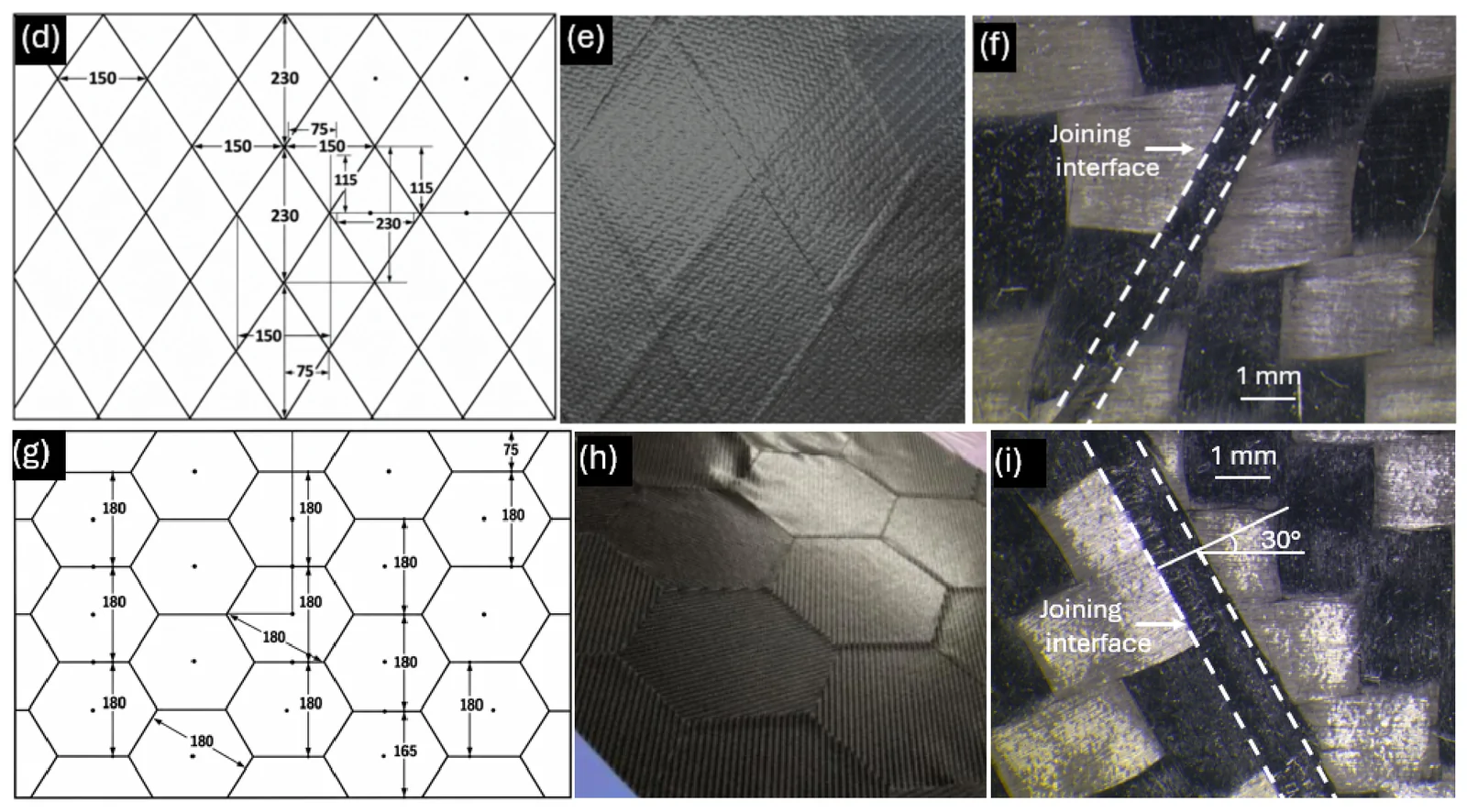
Schematic of patch configurations, corresponding manufactured laminates, and optical microscopic views of joining interfaces: (**a**–**c**) rectangular, (**d**–**f**) diamond, and (**g**–**i**) hexagonal arrangements. Dashed lines indicate the joining interfaces.

To investigate the effect of scrap geometry, the prepregs were cut into random rectangular, diamond, and hexagonal patches (see Figure 1a,d,g), and then assembled into larger plies for laminate manufacturing; see Figure 1b,e,h. The rectangular and diamond patches were automatically assembled with consistent fiber alignment, whereas the hexagonal patch assemblies exhibited fiber orientation mismatch of 30° at patch interfaces as a result of the rapid placement process, since meticulous fiber arrangement would require substantially more time; see Figure 1c,f,i. This approach reflects realistic industrial manufacturing conditions, in which practical production times must be taken into account. The joining interfaces are indicated by white dashed lines. The seam widths are approximately the same for all configurations, measuring about 0.7 mm.

The glass fiber and carbon fiber composite laminates, hereafter referred to as GFRP and CFRP, respectively, were manufactured using a 10-ply twill-woven prepreg layup, with all plies stacked in the same fabric orientation and the warp and weft directions aligned through the laminate thickness. During stacking, care was taken to prevent patch-boundary overlap, as such interfaces create resin-rich zones that can act as weak points in the structure. The laminates were cured by following the protocol: heating at 2 °C/min to 120 °C, holding under a pressure of 3 bar at 120 °C for 90 min, followed by cooling at 2 °C/min. The sandwich panels comprised a Rohacell^®^ foam core and four-ply skins on each side. For tensile and flexural testing, specimens were machined from the assembled laminates and sandwich panels, and the corresponding specimen dimensions are summarized in Table 2. Since all test specimens are narrower than 25 mm, which is considerably smaller than the seam length, each specimen contains a seam extending across its full width.

### 2.2. Mechanical Tests

Tensile tests were conducted in accordance with ASTM D3039 [24] to determine the tensile modulus and strength of the laminate specimens. The tensile strength was calculated using the formula as follows:

(1) $$\sigma_{t}=\frac{P}{\mathrm{bt}}$$

where *P* is the maximum load; *b* and *t* are the width and thickness of the specimens, respectively.

**Figure 2:**
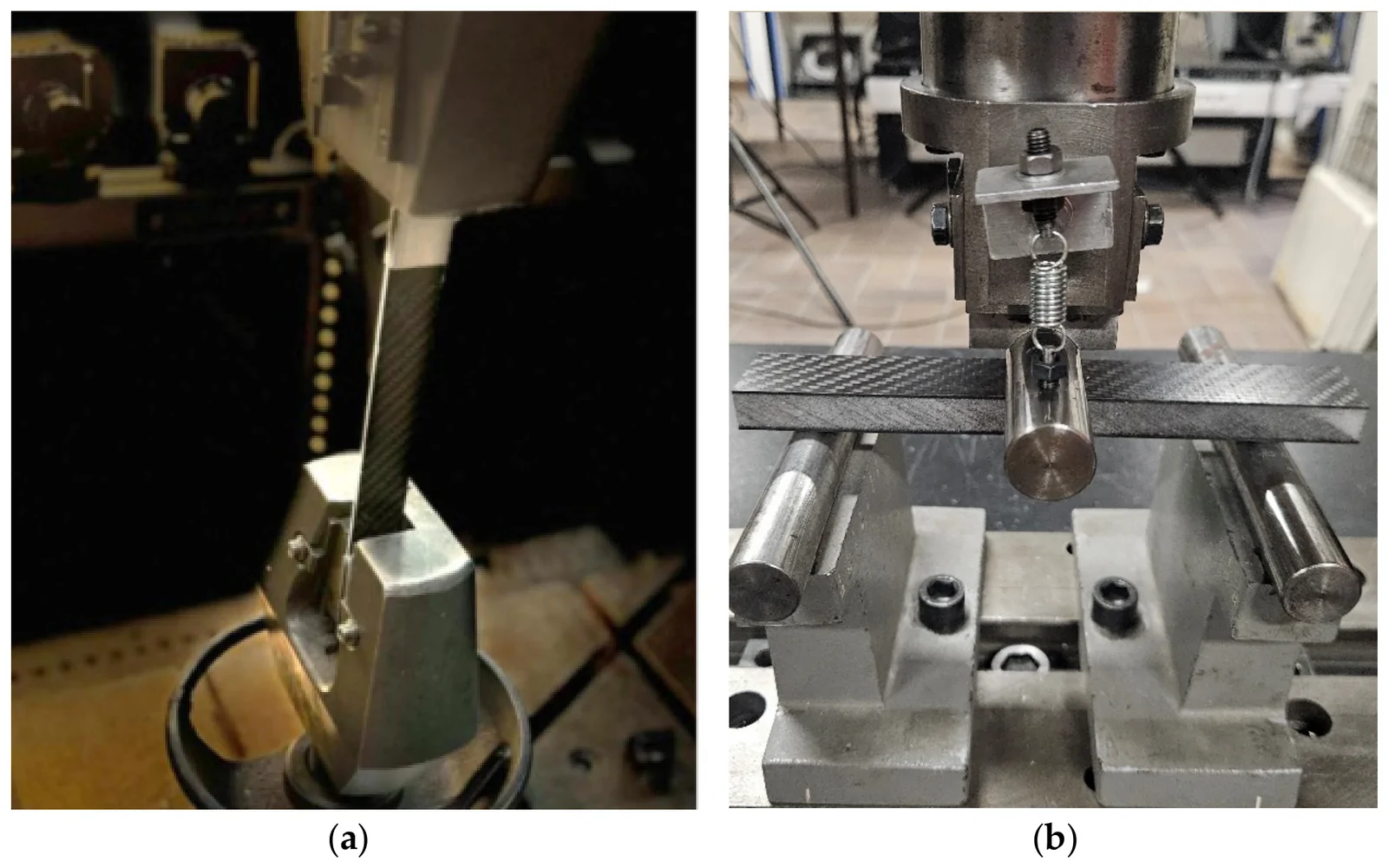
Mechanical tests on laminates and sandwich beams: (**a**) tensile tests and (**b**) three-point bending tests.

Aluminum end tabs with a thickness of 2 mm were bonded to both ends of the specimens to improve gripping and minimize stress concentrations during loading. All tests were performed using a universal testing machine (Instron 5982, provided by Instron Corporation, Norwood, MA, USA). A constant displacement rate of 2 mm/min was applied, and strain was measured using a 3D digital image correlation system provided by Correlated Solutions Europe (Kassel, Germany), as shown in Figure 2a.

Tensile testing of sandwich panels was initially considered; however, the specimens consistently failed by premature end crushing in the grip region before reaching meaningful tensile loads. This behavior is well documented for foam-core sandwich structures, in which the core lacks the compressive strength (1.5 MPa according to the manufacturer’s datasheet) required to transmit axial loads through the grips [25]. Additionally, sandwich structures are primarily designed to carry flexural loads rather than pure tensile loads. Hence, the tensile testing results for sandwich panels were disregarded, and tensile evaluation focused exclusively on the laminates.

Flexural behavior was assessed using three-point bending tests performed in accordance with ASTM D7264 [26] and ASTM C393 [27] for composite laminates and sandwich panels, respectively. The loading span was selected to satisfy a span-to-thickness ratio of 32:1 for laminates and 10:1 for sandwich panels. The same testing bench (Instron 5982) was employed for all flexural tests, with the load cell replaced by a 10 kN unit. The testing speed was 2 mm/min under displacement control. The flexural stress and strain were calculated by the following formulas:

(2) $$\sigma_{f}=\frac{3\mathrm{PL}}{2bt^{2}}$$

(3) $$\varepsilon_{f}=\frac{6\delta t}{L^{2}}$$

The peak stress of the stress–strain curves was taken as the flexural strength, and the flexural modulus of the laminates was calculated as the slope of the regression line over the segment between 0.1% and 0.3% strain on the stress–strain curves converted from the recorded load-displacement data.

For each geometry and material system, at least three specimens were tested, with the detailed number of replicates shown in Table 3.

### 2.3. Optical Microscopy

A Leica S8 APO stereomicroscope equipped with a DFC 295 digital camera (supplied by Leica Microsystems Wetzlar GmbH, Wetzlar, Germany) was employed to optically characterize the post-mortem failure mechanisms of both composite laminates and sandwich beams. Observations were conducted at a fixed magnification of 48×, which provided sufficient resolution to identify key damage features such as fiber fracture and interlaminar delamination.

## 3. Results and Discussion

### 3.1. Composite Laminates

#### 3.1.1. Tensile Properties

**Figure 3:**
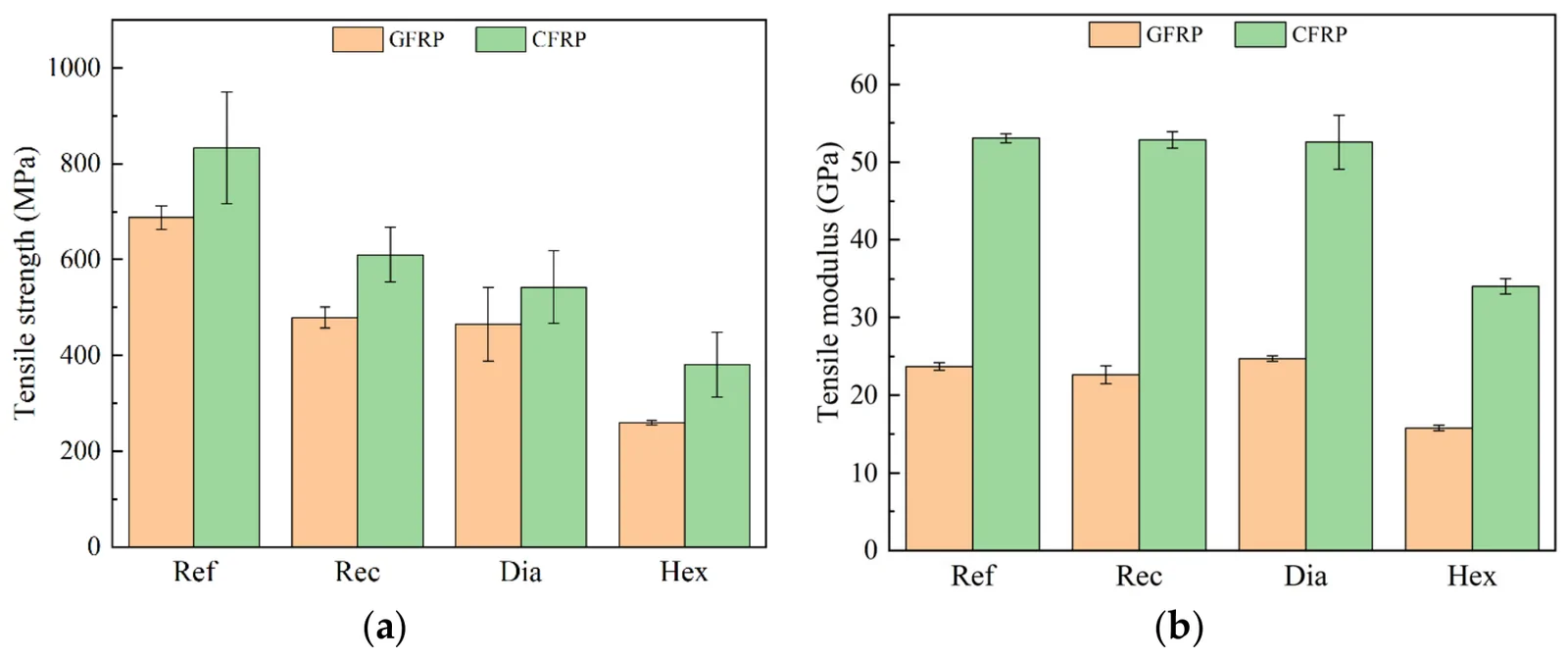
Tensile properties of scrap-assembled and reference composite laminates: (**a**) tensile strength and (**b**) tensile modulus. “Ref” denotes the reference (virgin) laminate, while “Rec”, “Dia”, and “Hex” correspond to laminates assembled from rectangular, diamond, and hexagonal scrap prepreg patches, respectively.

Figure 3 presents the tensile strength and tensile modulus of glass fiber and carbon fiber composite laminates manufactured using three different scrap patch geometries, benchmarked against the virgin reference laminates. All scrap-assembled glass fiber laminates exhibited a clear reduction in tensile strength, with decreases of 30% for the rectangular configuration, 32% for the diamond configuration, and 62% for the hexagonal configuration. A similar trend was observed for the carbon fiber laminates, showing strength reductions of 27%, 35%, and 54% for the rectangular, diamond, and hexagonal configurations, respectively. Pairwise statistical comparisons between each scrap-assembled configuration and the reference laminate were performed using a *t*-test. The resulting *p*-values were 3.8 × 10^−4^, 1.5 × 10^−2^, and 7.8 × 10^−6^ for the rectangular, diamond, and hexagonal configurations, respectively. All values were below the significance threshold of *p* = 0.05, indicating statistically significant differences in tensile strength between each scrap-assembled configuration and the reference laminate.

The observed strength degradation is primarily associated with the ply-joining boundaries introduced by the scrap assembly process. These interfaces interrupt local fiber continuity and stiffness, creating regions that may promote stress concentration and premature failure. Among the three scrap configurations, the rectangular patches exhibited the highest strength retention for both fiber systems, followed by the diamond configuration. The hexagonal configuration showed the poorest performance, which is attributed to the combined influence of its patch geometry and the corresponding seamline arrangement. In addition, local fiber misalignment observed only in the hexagonal configuration may have further contributed to its lower strength.

The tensile modulus shows markedly lower sensitivity to scrap geometry. For the rectangular and diamond configurations, the tensile modulus remained comparable to that of the virgin laminates, with *p*-values of 0.087 and 0.21, respectively. Since both values exceeded the significance threshold of 0.05, no statistically significant difference was observed, indicating that the initial tensile stiffness was largely preserved despite the presence of patch interfaces. This behavior can be explained by the fact that tensile modulus is measured at low strain levels (0.1–0.3%), where load transfer through resin-rich regions is still effective. The hexagonal configuration exhibits a noticeable reduction in tensile modulus, most likely caused by fiber orientation mismatch across adjacent patches, as the presence of off-axis patches reduces the effective modulus even within the elastic regime.

#### 3.1.2. Flexural Properties

**Figure 4:**
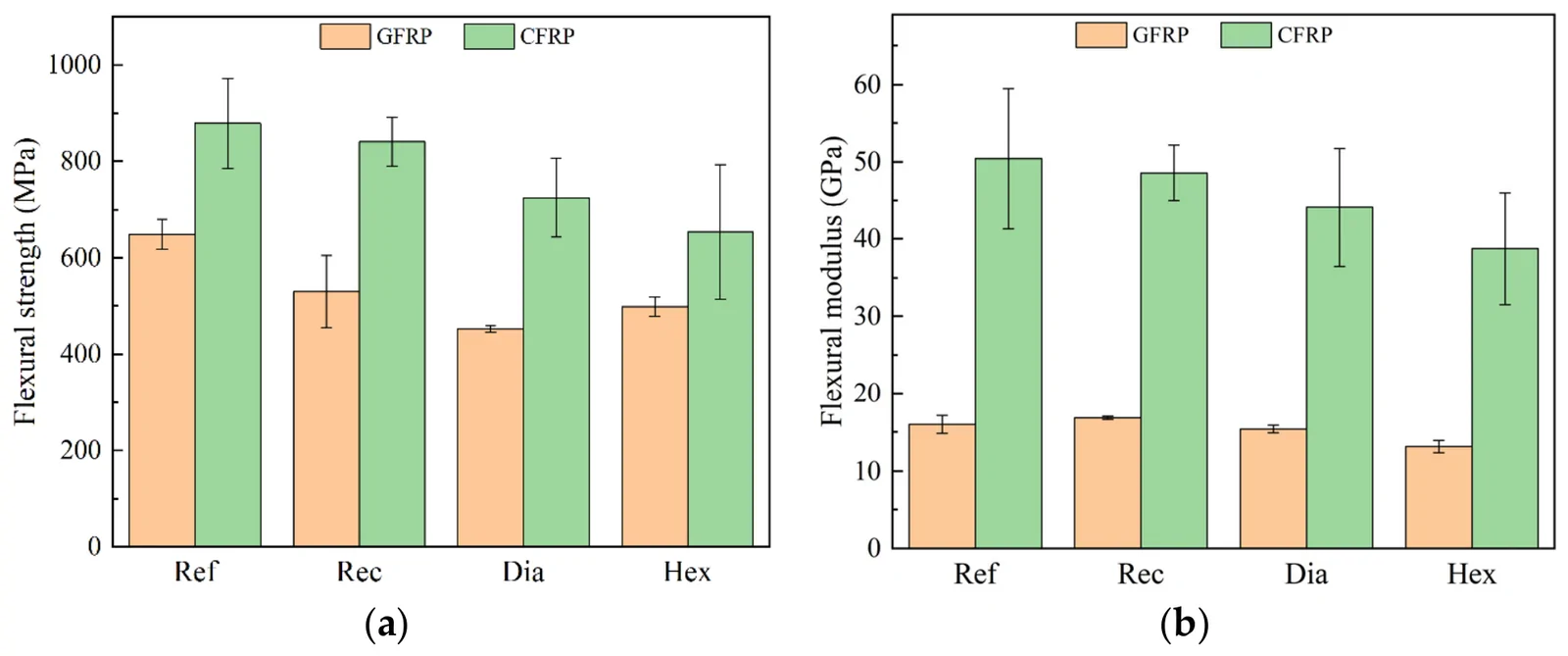
Flexural properties of scrap-assembled and reference composite laminates: (**a**) flexural strength and (**b**) flexural modulus.

The flexural strength and flexural modulus of scrap-assembled laminates with three different patch geometries are compared with the corresponding virgin laminates in Figure 4. While the virgin composites exhibit comparable strength under tensile and flexural loading, the scrap-assembled laminates show notably better strength retention under three-point bending than under uniaxial tension, particularly for the carbon fiber systems. The reductions in flexural strength are limited to 4% for the rectangular configuration, 18% for the diamond configuration, and 26% for the hexagonal configuration. The relatively large scatter in the flexural properties may partly originate from variations in seamline position among specimens. Because the seamline location was not controlled during specimen cutting, individual specimens contained seamlines at different positions relative to the bending midspan, resulting in different local stress states during testing.

The flexural strength of the rectangular configuration decreased by only approximately 4%, which is considerably smaller than the corresponding reduction in tensile strength. This difference can be rationalized by the distinct stress distributions under the two loading modes. During tensile loading, the gauge section experiences an approximately uniform axial stress, and joining interfaces within the load path can directly disturb fiber continuity and promote local stress concentrations. In bending, however, the longitudinal stress varies through the thickness, with the maximum tensile and compressive stresses occurring at the outer surfaces near the specimen midspan. Consequently, the effect of a seamline is expected to depend strongly on its position relative to the highly stressed outer regions and the bending midspan. Because the seamline position was not controlled during specimen preparation, some seamlines may have been located away from the most highly stressed region, thereby limiting their effect on the measured flexural strength. This interpretation should be regarded as a mechanical hypothesis, as the local stress redistribution around the joining interfaces was not directly quantified in the present study. Further finite-element analysis or full-field strain measurements would be required to establish the relationship between seamline position and flexural failure more rigorously.

For the flexural modulus, scrap-assembled glass fiber composites exhibit better stiffness retention than carbon fiber composites. This behavior is mainly attributed to the smaller modulus mismatch between glass fibers and the polymer matrix. Since glass fibers have a much lower elastic modulus than carbon fibers, local stiffness variations introduced by resin-rich interfaces or patch boundaries have a less pronounced influence on the global bending stiffness.

#### 3.1.3. Failure Mechanism Analysis of Laminates

**Figure 5:**
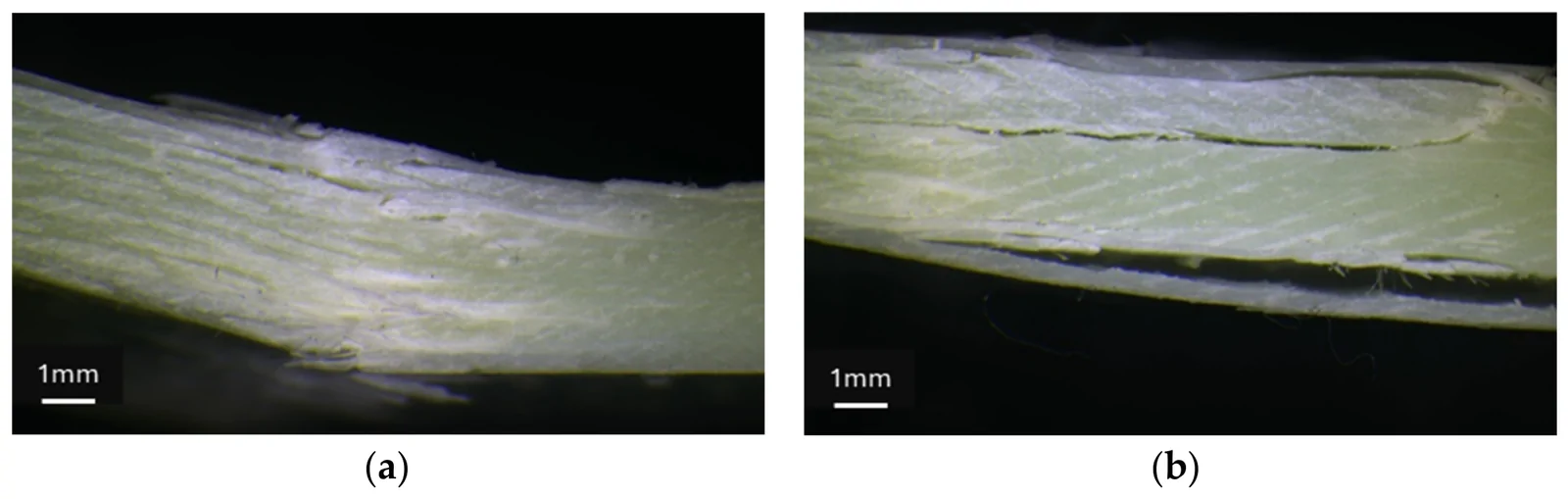

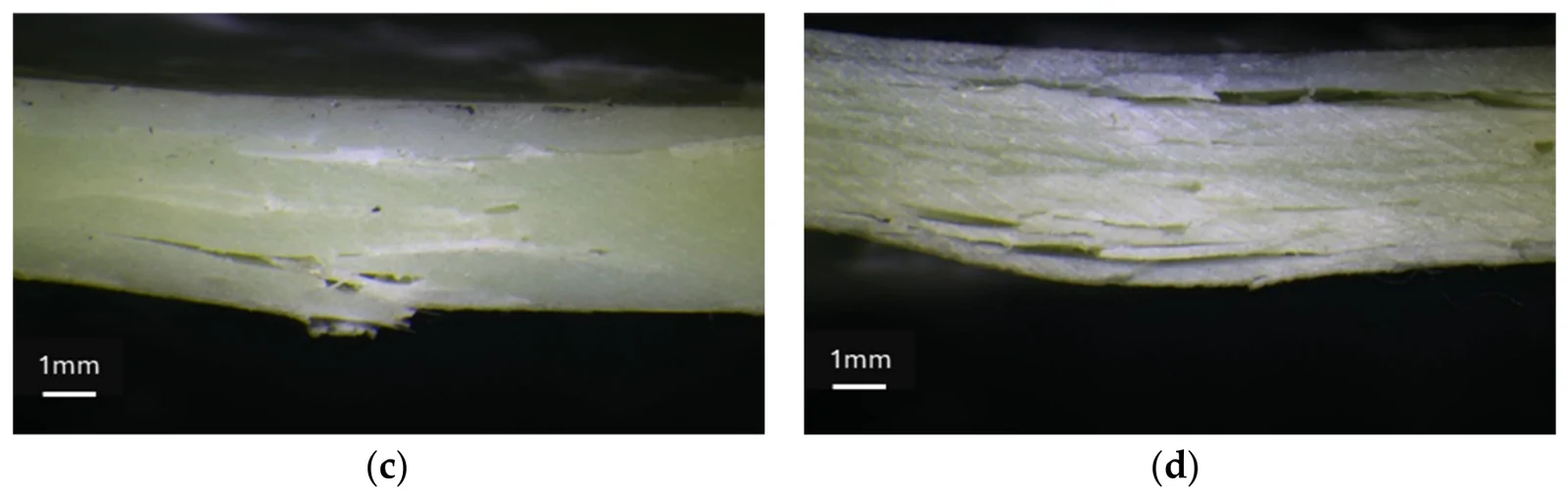
Fracture morphology of virgin and scrap-assembled glass fiber composite laminates after three-point bending: (**a**) reference laminate, (**b**) rectangular scrap-assembled laminate, (**c**) diamond scrap-assembled laminate, and (**d**) hexagonal scrap-assembled laminate.

The fracture morphology of virgin and scrap-assembled glass and carbon fiber composites under three-point bending was assessed using an optical microscope. Figure 5 presents side views of all GFRP samples. The scrap-assembled laminates show more extensive delamination than the virgin laminates. This behavior is attributed to the resin-rich ply-joining interfaces of the scrap-assembled laminates, which are likely to experience elevated interlaminar shear and peel stresses under flexural loading and are therefore more susceptible to delamination initiation. In addition, the consolidation quality at patch interfaces may be inferior to that of continuous plies due to non-uniform resin distribution, further reducing interlaminar integrity. As a result, delamination develops more readily in scrap-assembled laminates, contributing to the observed reduction in flexural strength and flexural modulus compared with the virgin samples.

**Figure 6:**
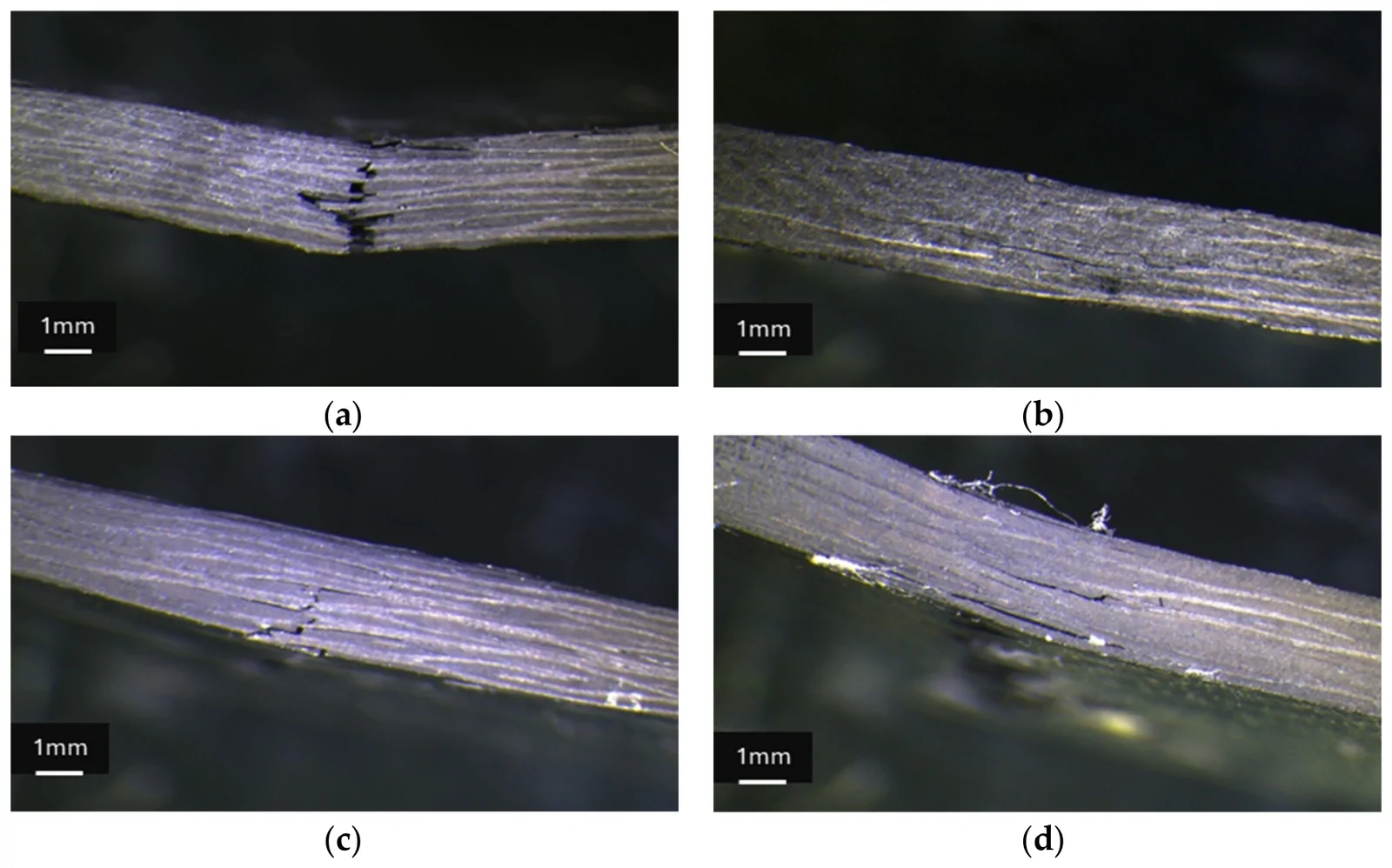
Fracture morphology of virgin and scrap-assembled carbon fiber composite laminates after three-point bending: (**a**) reference laminate, (**b**) rectangular scrap-assembled laminate, (**c**) diamond scrap-assembled laminate, and (**d**) hexagonal scrap-assembled laminate.

The carbon fiber systems show slightly different fracture behaviors. The virgin laminate shows only minor delamination, with fiber breakage dominating the failure mode, as shown in Figure 6a. In contrast, the rectangular scrap-assembled laminate exhibits more pronounced delamination, accompanied by less evident fiber fracture. For the diamond and hexagonal configurations, delamination migration is observed, with delamination extending into neighboring plies via ply-through matrix cracking. This behavior is likely associated with the presence of off-axis seamlines in these configurations, which induce higher local shear stresses and thereby facilitate the development of through-thickness matrix cracking [28,29].

To further quantify the observed damage, the number of visible delaminations was counted from the optical micrographs, as summarized in Table 4. For GFRP, the scrap-assembled configurations generally exhibited a greater number of delaminations than the reference, with the hexagonal configuration showing the highest count. A similar trend was observed for CFRP, where the diamond and hexagonal configurations exhibited more delamination than the reference. These results support the qualitative microscopy observations that the scrap-assembled interfaces promote more distributed interlaminar damage. The measured delamination lengths are also reported in Table 4. However, they are not used as the primary basis for comparison because the delamination extent can depend on the crosshead displacement and the progression of damage during the three-point bending test.

It should be noted that the failure mechanisms observed in this study correspond to quasi-static loading conditions at a displacement rate of 2 mm/min. Loading rate can influence matrix-dominated and interface-related damage processes, including matrix cracking, interfacial debonding, and delamination, and may therefore alter the relative contribution of different failure modes. Since loading-rate effects were not investigated here, the observed fracture behavior should be interpreted specifically within the quasi-static test conditions adopted in this study.

### 3.2. Sandwich Panels

#### 3.2.1. Flexural Load Capacity

**Figure 7:**
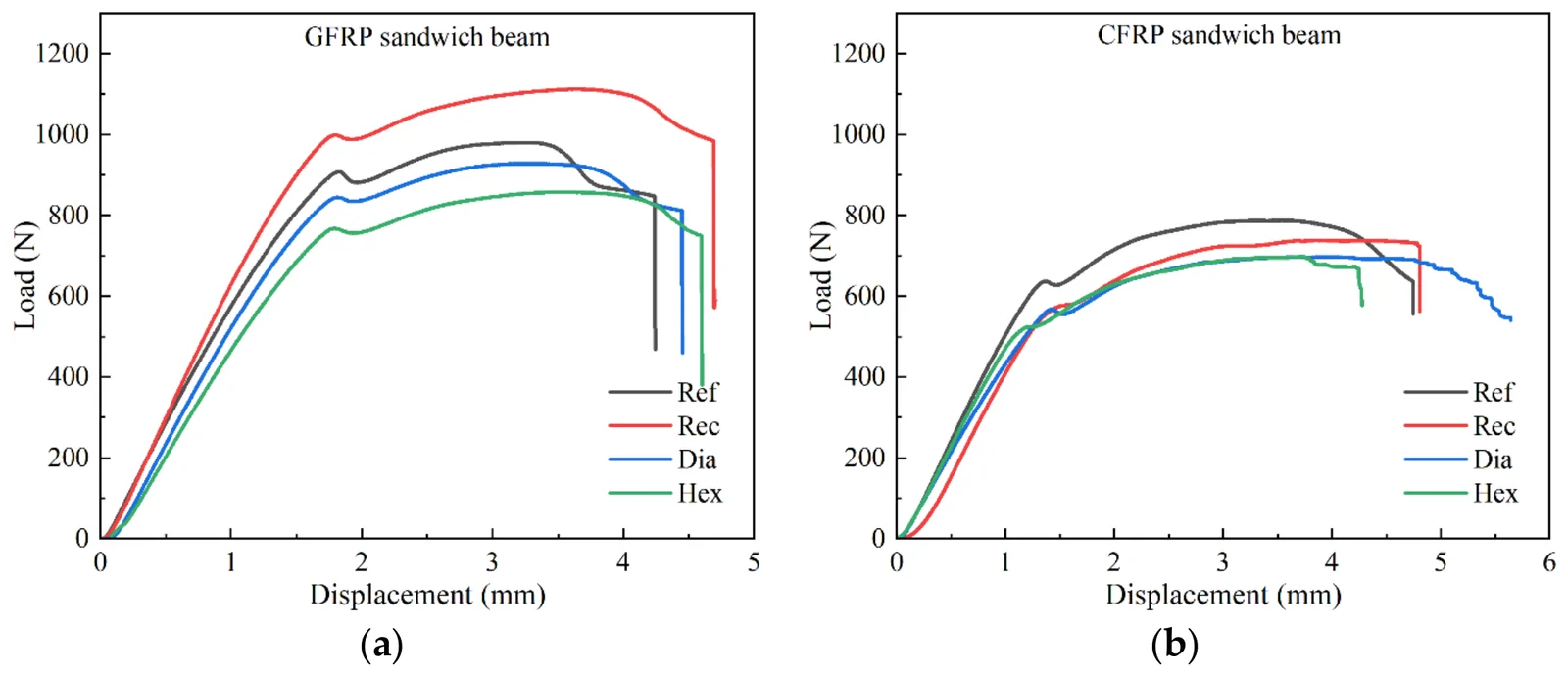
The load-displacement curves of sandwich beams: (**a**) GFRP and (**b**) CFRP.

The load-displacement curves of GFRP and CFRP sandwich beams are shown in Figure 7a and Figure 7b, respectively. Overall, the load capacity (maximum load) of the scrap-assembled sandwich beams follows the same general trend as the flexural strength measured for their corresponding composite laminates. An exception is observed for the rectangular scrap-assembled GFRP sandwich beam, which exhibits a higher load capacity than the virgin sandwich beam. However, this load-based comparison is influenced by the dimensional differences between the specimens. The virgin GFRP sandwich beams have an average total thickness (skin + core) of 13.7 mm, whereas the rectangular scrap-assembled sandwich beams exhibit an increased thickness of approximately 14.3 mm. Since the skins are only approximately 2 mm thick compared with the 11.5 mm foam core, this difference represents only a modest change in the overall sandwich thickness. Nevertheless, even a small increase in skin thickness and skin separation can increase the bending stiffness and load required to reach failure.

A simple normalization of the maximum load by the total specimen width and thickness did not alter this trend, with the rectangular configuration still showing a higher normalized value. This is expected because the small increase in skin thickness produces only a limited change in the total sandwich dimensions. Therefore, such a simple normalization does not fully account for the mechanics of the sandwich structure.

Although other scrap-assembled sandwich panels with different patch geometries also exhibited increased thickness compared with the virgin specimens, their load capacities did not show a similar improvement. This suggests that the influence of geometry is counteracted by the greater reduction in the intrinsic strength of the corresponding scrap-assembled laminates. Consequently, the stiffness and load-bearing gains introduced by geometric thickening are insufficient to fully compensate for the reduction in intrinsic laminate strength for these geometries.

**Figure 8:**
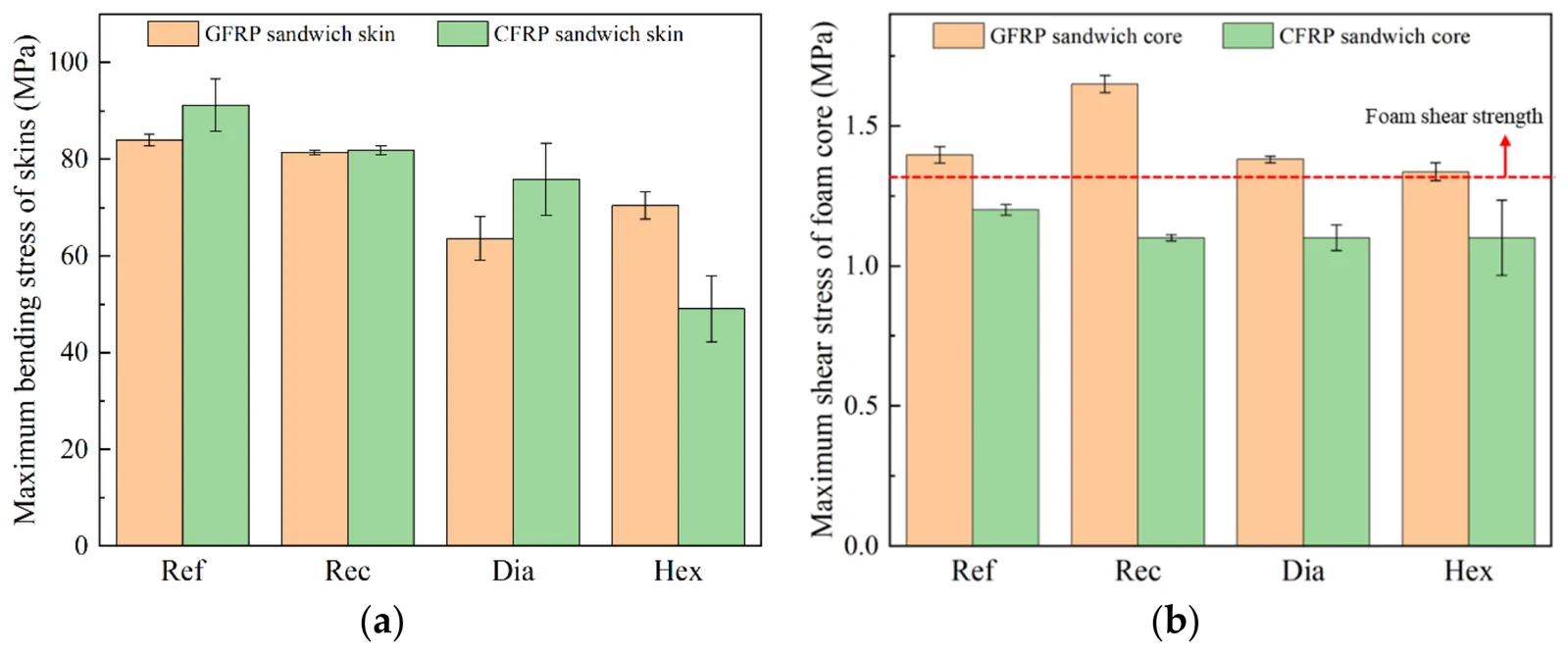
Comparison of GFRP and CFRP sandwich beams with different scrap geometries in terms of (**a**) maximum skin bending stress and (**b**) maximum foam-core shear stress.

The lower load capacity measured for the CFRP sandwich beams compared to the GFRP sandwich beams is again attributed to their slightly reduced overall thickness (~13.1 mm for CFRP). To account more rigorously for the influence of sandwich geometry, the maximum bending stress in the skin and the maximum shear stress in the core under three-point bending were calculated according to ASTM C393, using the dimensions of both the skin and the core, as shown in Figure 8. It is observed that the maximum bending stress of the GFRP face sheets is lower than that of the CFRP face sheets, which differs from the trend observed in the load-based results. Similarly, although the rectangular scrap-assembled GFRP sandwich beam exhibits a higher load capacity than the virgin specimen, it shows a lower maximum bending stress in the face sheet at failure. This geometry-corrected comparison therefore indicates that the apparent advantage of the rectangular configuration in terms of maximum load originates mainly from dimensional effects rather than superior intrinsic flexural performance.

The measured maximum bending stress in the face sheets and the shear stress in the foam core can be further used to evaluate the failure mechanisms of the sandwich beams. The maximum bending stresses in both GFRP and CFRP face sheets are significantly lower than the flexural and tensile strengths of their corresponding laminates (see Figure 3a and Figure 4a), indicating that failure of the sandwich beams was not caused by face-sheet fracture. When compared with the shear strength of the foam core, ROHACELL^®^ 71WF (1.3 MPa, as provided by the manufacturer Evonik), the maximum core shear stresses calculated in all the GFRP sandwich beams exceed this value, confirming that failure in the GFRP sandwich beams was governed by foam-core shear. In contrast, for the CFRP sandwich beams, both the calculated face-sheet bending stress and the core shear stress remain below their respective strength limits, suggesting that the failure mechanism cannot be explained by strength-based criteria alone.

#### 3.2.2. Failure Mechanism Analysis of Sandwich Beams

**Figure 9:**
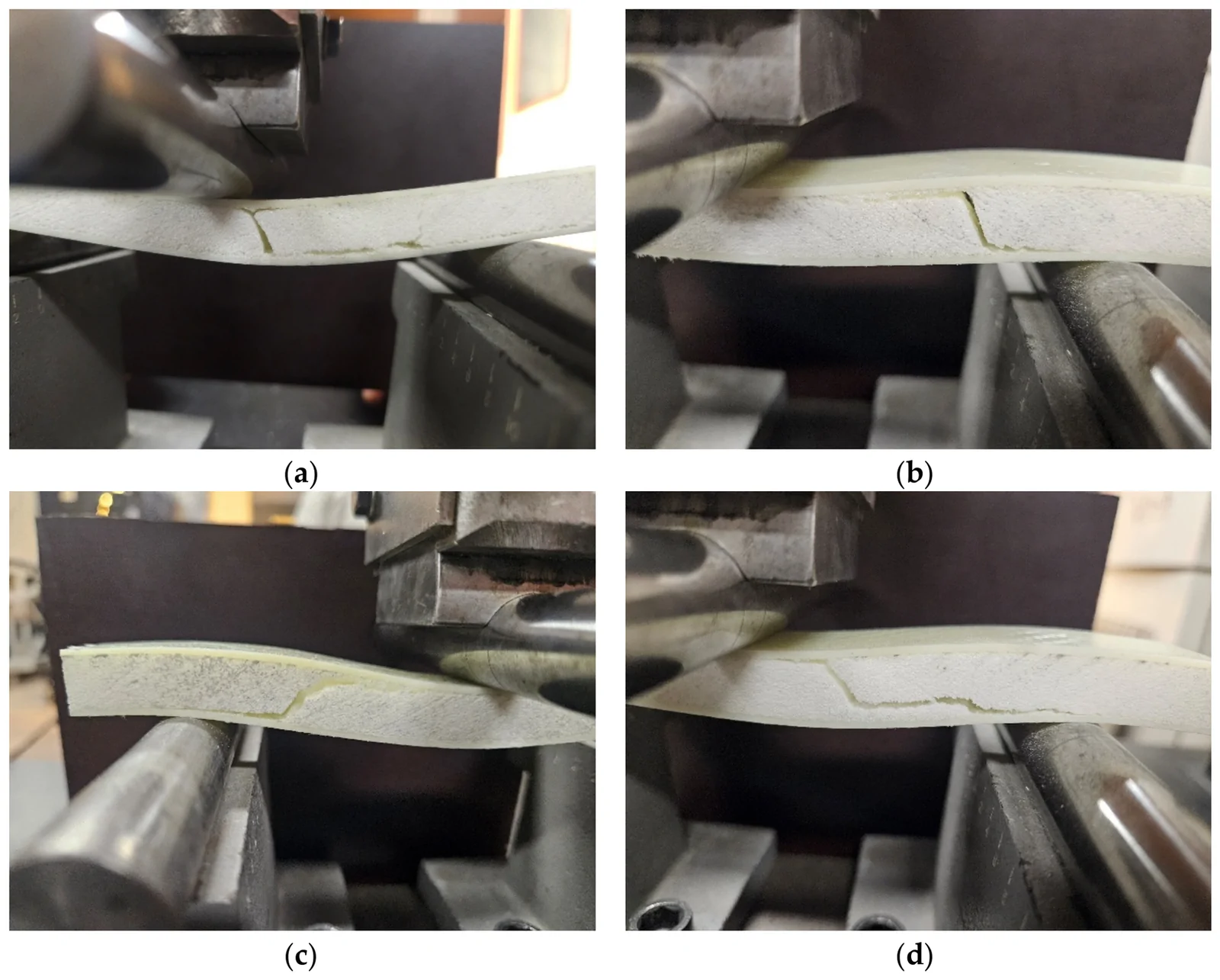
Failure morphology of GFRP sandwich beams under three-point bending: (**a**) reference sandwich beam, (**b**) rectangular scrap-assembled sandwich beam, (**c**) diamond scrap-assembled sandwich beam, and (**d**) hexagonal scrap-assembled sandwich beam.

The failure mechanisms of both GFRP and CFRP sandwich beams were further investigated through optical analysis. For the GFRP sandwich beams, all specimens exhibited a consistent collapse sequence characterized by core shear failure, followed by skin–core interface debonding, as shown in Figure 9. No significant damage was observed in the GFRP face sheets themselves. This observation is in good agreement with the stress-based analysis, which shows that the maximum bending stress in the GFRP face sheets remains well below the flexural and tensile strengths of the corresponding laminates, while the calculated core shear stress exceeds the shear strength of the foam core. In addition, the relatively low span-to-thickness ratio of 10 enhances the contribution of transverse shear, further promoting shear-dominated failure.

**Figure 10:**
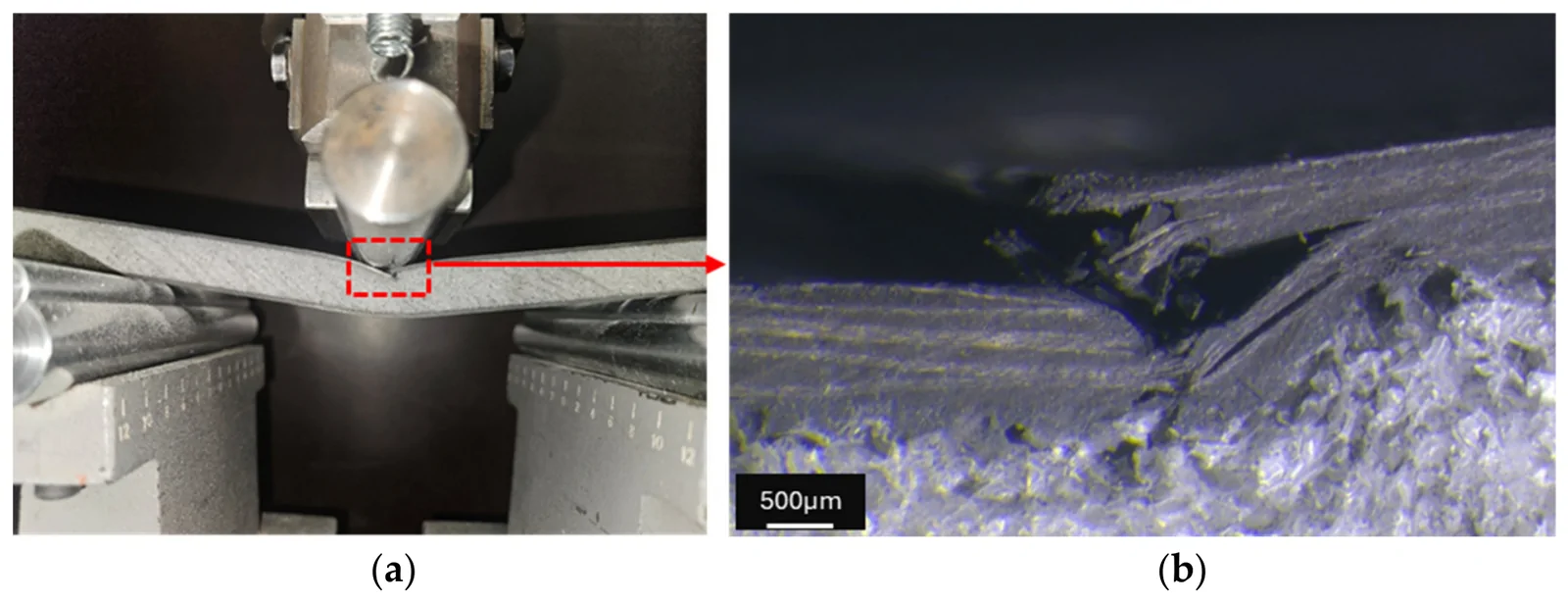
Failure morphology of CFRP sandwich beams: (**a**) overview of the failure and (**b**) zoomed-in view of the local skin buckling.

The CFRP sandwich beams exhibited a different failure behavior. Although inclined shear bands were still visible within the foam core, no complete core shear fracture was observed (see Figure 10a). This indicates that the core shear stress remained below its critical value at failure, which is consistent with the measured core shear stress being lower than the core shear strength. The dominant damage mode was characterized by local deformation of the upper skin under compressive loading, consistent with skin wrinkling or local buckling (see Figure 10b). Since this deformation occurred while the calculated skin stress remained below the flexural strength of the CFRP laminate, conventional material-strength failure is less likely to be the governing mechanism. The observed local instability may be related to the interaction between the relatively stiff CFRP skin and the compliant foam core, which can promote localized deformation of the compressed skin. However, a dedicated wrinkling or buckling analysis would be required to confirm elastic instability quantitatively [30].

The observed sandwich-beam failures are consistent with established sandwich mechanics, where the governing mode depends on the relative properties of the skin and core. Previous studies have reported core shear, skin/core debonding, and face-sheet instability as common failure modes under bending [31,32]. Accordingly, the core shear followed by debonding observed in the GFRP beams is consistent with prior findings, while the localized compressive-face deformation in the CFRP beams is consistent with local skin instability.

## 4. Demonstrator Testing

**Figure 11:**
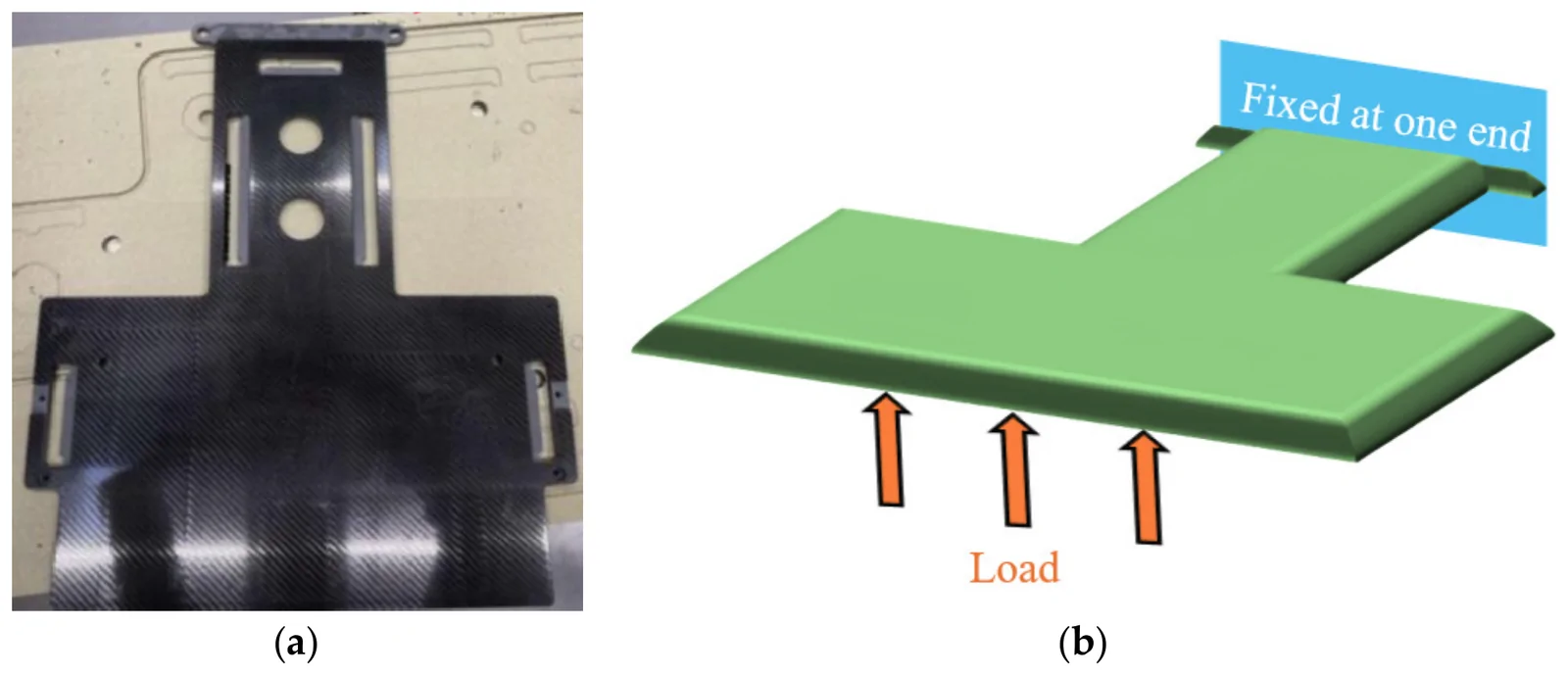
(**a**) A head–neck board demonstrator and (**b**) loading scenario.

Based on the evaluation of the tensile and flexural performance of both composite laminates and sandwich beams, the rectangular configuration consistently demonstrated the best mechanical performance among the three investigated geometries. Consequently, this configuration was selected for the fabrication of a demonstrator, namely a head–neck board for radiotherapy treatment of cancer patients, as shown in Figure 11a. The demonstrator is an irregularly shaped sandwich panel incorporating four layers of carbon fiber composite laminates as face sheets.

The typical loading scenario for the head–neck board involves bending loads applied by a nurse’s hand when lifting the free end of the board after treatment, while the opposite end remains fixed to the machine frame if the fixture is not released, as illustrated in Figure 11b. In the absence of a dedicated standard for this application, the head–neck board was evaluated against a required load of 400 N, based on an internal Orfit Industries design criterion consisting of an estimated service load of 100 N and a safety factor of 4.

**Figure 12:**
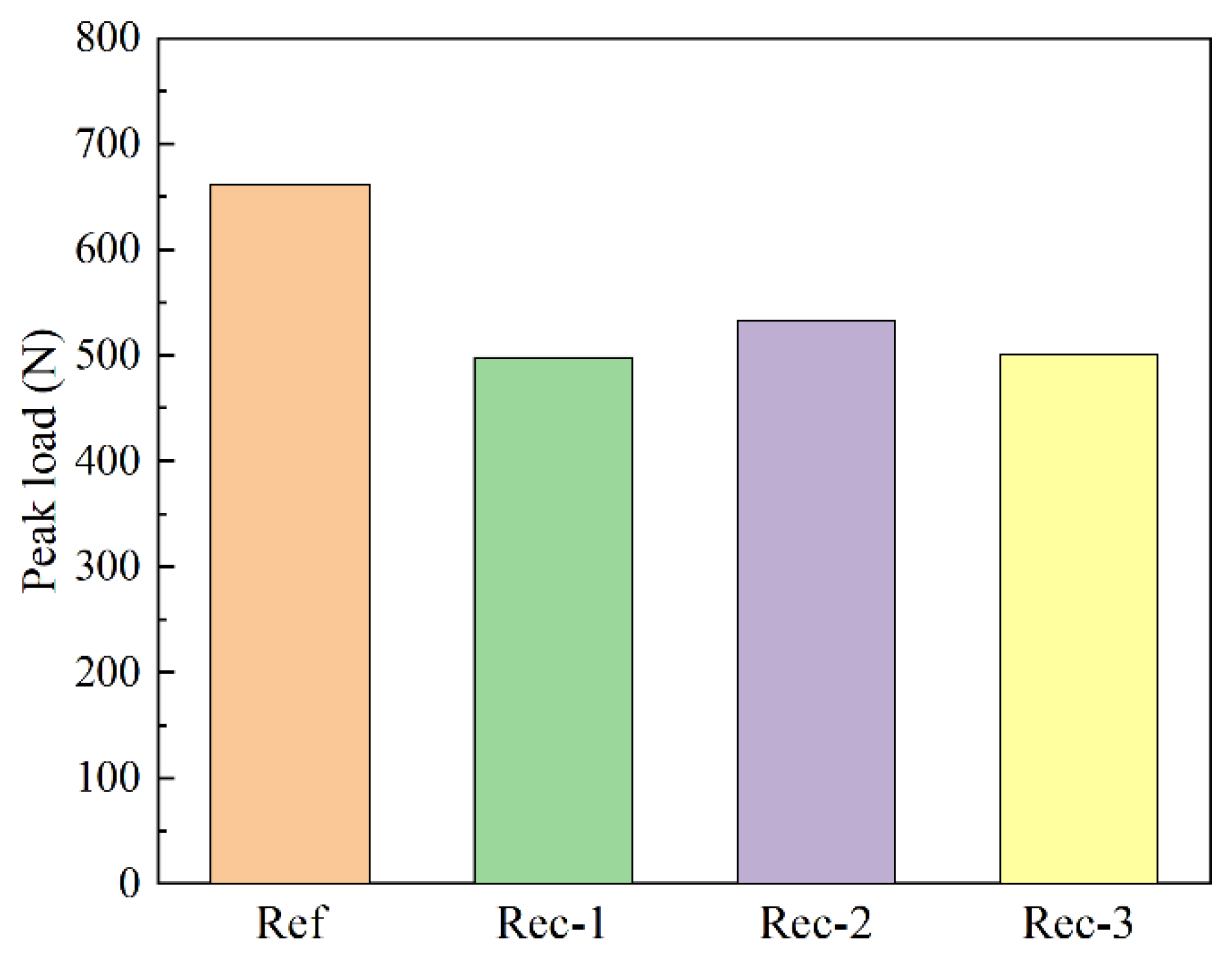
Comparison of load capacity of reference and rectangular scrap-assembled demonstrator.

To evaluate the bending resistance of the rectangular scrap-assembled board, the panel was inverted, fixed at one end using bolts, and loaded downward at the free end. Figure 12 compares the measured load capacity of three individual rectangular scrap-assembled boards with that of a commercial reference board manufactured on the production line of Orfit Industries. While the reference board exhibited a load capacity of 660 N, the rectangular scrap-assembled board achieved a mean load capacity of 510 N after three repetitions, corresponding to a bending capacity retention of 77%. Most importantly, the retained bending performance exceeds the required criterion of 400 N, thereby demonstrating the feasibility of reusing and upcycling uncured prepreg scraps for functional composite products.

## 5. Conclusions

This study systematically investigated the mechanical performance and failure behavior of composite laminates and sandwich beams manufactured from uncured prepreg scraps with different patch geometries. Based on the experimental results and analysis, the following conclusions can be drawn:

(1) Scrap-assembled laminates showed a reduction in tensile strength compared with virgin laminates, primarily due to patch-joining interfaces that create resin-rich regions and local stiffness discontinuities. However, the tensile modulus was largely retained for rectangular and diamond configurations, indicating that elastic properties at low strain levels are less affected by scrap-induced interfaces.
(2) Flexural strength was retained significantly better than tensile strength in scrap-assembled laminates, particularly for carbon fiber systems. This is attributed to the localized stress state in bending and the lower probability of seamlines intersecting the critical midspan region. Rectangular patches consistently showed the best performance, while hexagonal patches experienced fiber orientation mismatch and poorer strength retention.
(3) GFRP sandwich beams failed mainly by foam-core shear, followed by skin–core debonding, in agreement with stress-based predictions. In contrast, CFRP sandwich beams failed by local skin wrinkling under compressive loading, governed by elastic instability rather than material strength.
(4) A head–neck board demonstrator made from rectangular scrap-assembled CFRP sandwich panels retained 77% of the bending capacity of a commercial reference board and exceeded the required safety load. This confirms the feasibility of upcycling uncured prepreg scraps into functional, load-bearing composite products.

Overall, the findings demonstrate that uncured prepreg scraps, particularly when assembled using rectangular patch geometries, offer a structurally viable route for circular composite manufacturing. The insights from this work provide guidance on design and failure mechanisms, supporting the broader industrial adoption of prepreg scrap upcycling.

Several limitations of this work should be noted. While the influence of patch geometry on tensile, flexural, and sandwich beam performance was identified, the underlying mechanisms linked to local microstructural characteristics of the joining interfaces, including void content, resin-rich-zone dimensions, and local fiber volume fraction, were not quantitatively characterized. Further characterization, for example, using X-ray micro-computed tomography, would be required to establish direct relationships between local interface morphology and the observed mechanical response. Another limitation is that the seamline position relative to the specimen midspan was not controlled during specimen preparation. This may have contributed to the scatter in the measured flexural properties. Future studies should systematically control or record seamline position to quantify its effect on flexural performance.

## Figures and Tables

**Table 1:** Material properties of the GFRP and CFRP prepregs.

Material	Fiber Areal Weight	Resin Content	Cured Ply Thickness
GFRP	290 g/m^2^	46%	0.26 mm
CFRP	220 g/m^2^	48%	0.19 mm

**Table 2:** Dimensions of laminates and sandwich beams for tensile and flexural tests.

Testing Type	Laminates	Sandwich Beams
Tensile	250 mm × 25 mm × 2.5 mm	250 mm × 25 mm × 13.5 mm (foam core: 11.5 mm; skins: 1 mm)
Three-point bending	100 mm × 25 mm × 2.5 mm	190 mm× 25 mm × 13.5 mm (foam core: 11.5 mm; skins: 1 mm)

**Table 3:** Number of replicates for each material system.

Material	Test	Reference	Rectangular	Diamond	Hexagonal
GFRP laminate	Tensile	3	3	3	3
CFRP laminate	Tensile	5	4	6	6
GFRP laminate	Bending	3	3	3	3
CFRP laminate	Bending	6	7	7	6
GFRP sandwich	Bending	3	3	3	3
CFRP sandwich	Bending	3	3	3	3

**Table 4:** Number and measured length of delamination in GFRP and CFRP laminates.

Material System	Patch Geometry	Number of Delamination	Delamination Length (mm)
GFRP	Reference	2	3.5 ± 0.5
	Rectangular	3	12.1 ± 1.0
	Diamond	3	3.4 ± 2.0
	Hexagonal	6	5.6 ± 4.6
CFRP	Reference	2	1.2 ± 0.5
	Rectangular	4	2.9 ± 2.1
	Diamond	5	1.4 ± 0.4
	Hexagonal	6	2.4 ± 2.1

## Data Availability

The original contributions presented in the study are included in the article, further inquiries can be directed to the corresponding author.
